# What Research Institutions Can Do to Foster Research Integrity

**DOI:** 10.1007/s11948-020-00178-5

**Published:** 2020-01-21

**Authors:** Lex Bouter

**Affiliations:** 1 Department of Epidemiology and Biostatistics, Amsterdam University Medical Centers, Amsterdam, The Netherlands; 2 Department of Philosophy, Faculty of Humanities, Vrije Universiteit, Amsterdam, The Netherlands

**Keywords:** Research integrity, Research misconduct, Fabrication, Falsification, Questionable research practices, Meta-research

## Abstract

In many countries attention for fostering research integrity started with a misconduct case that got a lot of media exposure. But there is an emerging consensus that questionable research practices are more harmful due to their high prevalence. QRPs have in common that they can help to make study results more exciting, more positive and more statistically significant. That makes them tempting to engage in. Research institutions have the duty to empower their research staff to steer away from QRPs and to explain how they realize that in a Research Integrity Promotion Plan. Avoiding perverse incentives in assessing researchers for career advancement is an important element in that plan. Research institutions, funding agencies and journals should make their research integrity policies as evidence-based as possible. The dilemmas and distractions researchers face are real and universal. We owe it to society to collaborate and to do our utmost best to prevent QRPs and to foster research integrity.

## Introduction

Traditionally research integrity has focussed on the prevention, identification and handling of the three deadly sins of scientific and scholarly research: fabrication, falsification and plagiarism (National Academies of Sciences 2017). In many countries the attention for research integrity was fuelled by a misconduct case that got a lot of media exposure. In The Netherlands it was the Diederik Stapel case (Levelt, Noort and Drenth Committees 2012) that served as a call to arms. It shocked many within and outside Academia but turned out to be a blessing in disguise as well. Stapel’s successor as dean of the faculty of social sciences of Tilburg University acted according to the dictum ‘never waste a good crisis’ (Sijtsma 2017). The other Dutch universities followed and local and national measures were taken. This sequence of events seems typical for many countries.

In recent years attention has shifted to the lesser breaches of research integrity that are commonly referred to as questionable research practices or QRPs (Bouter et al. 2016; Haven et al. 2019). The idea is that these are much more prevalent and thus collectively do more harm to the validity of and the trust in the results of research (National Academies of Sciences 2017; Editorial 2019; Macleod and Mohan 2019). Examples are selective reporting, P-hacking, and hypothesising-after-the-results-are-known or HARK-ing. In an excellent paper from the Meta-Research Center of Tilburg University 34 QRPs are identified as researcher degrees of freedom that should be avoided in hypothesis-testing research (Wicherts et al. 2016). QRPs have in common that they can help to make study results more exciting, more positive and more statistically significant, which in its turn increases the likelihood to be accepted by a high impact journal, to get many citations, and to obtain the next grant or academic tenure.

Almost all researchers want to deliver good quality science, to avoid QRPs, and to follow their moral compass to steer a course of research integrity. Like any compass the functioning of a moral compass depends on its quality and on external factors. The quality is determined by the virtuousness of the individual at issue. Major external factors that can corrupt the moral compass concern the local research climate and the perverse incentives of the science system as a whole. Researchers need help from their research institution in optimising the functioning of their moral compass. That help involves adequate education and skills training, good facilities and expert help, and clear codes and procedures. That being said we should realize that research institutions experience perverse incentives that concern the way research is financed and evaluated by governments and research funders which ultimately trickle down the researchers themselves (Anderson 2019; Bagioli et al. 2019). Consequently research institutions need help from the other stakeholders in the research system (Bouter 2018).

## Duties of Care

The Netherlands Code of Conduct for Research Integrity specifies 61 standards for good research that mirror in fact as many QRPs to be avoided (Netherlands code of conduct on research integrity 2018). An unique feature of the code is that it also contains a chapter on the duties of care research institutions have to empower their research staff to steer away from QRPs. This idea is not new and was already contained in the Singapore Statement (2010) that says in responsibility 13: ‘Research institutions should create and sustain environments that encourage integrity through education, clear policies, and reasonable standards for advancement, while fostering work environments that support research integrity’. In other words: research institutions need to have a Research Integrity Promotion Plan. The Horizon 2020 funded consortium Standard Operating Procedures for Research Integrity (SOPs4RI 2020) will offer research institutions help to formulate this plan. Having implemented such a plan might become a contractual obligation for institutions accepting grants from the next EU framework program Horizon Europe.

The idea is that the Research Integrity Promotion Plan explains what the research institution sets out to do—in the context of its mission, disciplinary focus and type of research it performs—to promote research integrity. The plan needs to cover a set of mandatory topics and will typically describe a mix of education programs, codes, manuals, policy measures, regulations, facilities, audit schemes, and support systems. SOPs4RI will produce a toolbox filled with Standard Operating Procedures (SOPs) and guidelines that can help research institutions to formulate their Research Integrity Promotion Plan (e.g. ORI 1995; ENRIO 2020; Forsberg et al. 2018; Penders et al. 2018). A preliminary version of the SOPS4RI toolbox will become available by the end of 2020 and the final version will be ready in 2022. The difference between a SOP and a guideline is gradual, with SOPs being more strict step-by-step recipes and guidelines offering some freedom of choice. It’s important to make this not another box ticking exercise, but to ensure that researchers appreciate and use the guidance offered by their institution.

Initiatives of research institutions and other stakeholders to improve the quality of research and research integrity are by no means unique to The Netherlands. Some important initiatives are the USA Centre for Open Science (COS 2020), the UK Reproducibility Network (UKRN 2020), the European Quality In Preclinical Data Innovative Medicine Initiative (EQIPD IMI 2020), and the German Quality, Ethics, Open Science, Translation Center (QUEST 2020). Taken together currently there is a lack of solid guidance for research institutions that want to improve responsible research practices. The examples are scattered and not all evidence-based and fit for application. The hope is that the results of the SOPs4RI consortium will improve this situation substantially.

## Perverse Incentives

Arguably one of the most important things research institutions should do is to avoid perverse incentives in assessing researchers for career advancement. The current dominant focus on bibliometric indicators derived from publication and citation counts sends a strong message that only these things really matter (Moher et al. 2018). During recent years the myopic use of quantitative indicators in research evaluations has been criticised. This led to initiatives like the Leiden Manifesto (Hicks et al. 2015) and the San Francisco Declaration on Research Assessment (DORA 2020). In line with this the Hong Kong Principles for assessing researchers (Moher et al. 2019) were formulated and endorsed at the 6th World Conference on Research Integrity (WCRIF 2020). These principles will help research institutions that adopt them to minimise perverse incentives that invite to engage in questionable research practices or worse.

The Hong Kong Principles are chosen with a view to explicitly recognise and reward researchers for behaviour that leads to trustworthy research by avoiding QRPs. The principles have been developed with the idea in mind that their implementation could assist in how researchers are assessed for career advancement with a focus on behaviours that strengthen research integrity. Five principles were formulated: assess responsible research practices, value complete reporting, reward the practice of open science, acknowledge a broad range of research activities, and recognise essential other tasks like peer review and mentoring. For each principle a rationale for its inclusion is provided and examples of research institutions where these principles are already being adopted are given.

## Meta-research

The little empirical evidence on interventions to improve responsible research practices we have is often of poor quality, negative or both. This is illustrated by the Cochrane review that summarizes the evidence on interventions to prevent misconduct and promote integrity in research and publication (Marusic et al. 2016). Research institutions should make their research integrity policies as evidence-based as possible. In hindsight it’s difficult to understand why it took us so long to establish a solid tradition in research on research integrity. That only started to happen recently and was fuelled by granting programs like the Horizon 2020 Science with and for Society (SwafS) calls for research ethics and research integrity (EC 2020). In the Netherlands the programs on Fostering Responsible Research Practices (ZonMw 2020) and Replication Studies (NWO 2020) contributed to the emerging field of research on research. At the 5th World Conference on Research Integrity the Amsterdam Agenda was adopted that strongly encourages research on research integrity especially focusing on solutions that really work and effect change (Amsterdam Agenda 2015; Mayer et al. 2017).

That being said there is still a lot we don’t know about research integrity in research institutions. To fill this gap in May 2020 all researchers in Dutch universities and university medical centres will be invited to participate in the National Survey on Research Integrity. The survey is expected to provide valid and reliable knowledge on how often specific QRPs occur and what their underlying explanatory variables are. This will provide insights that help research institutions to improve their policies and to fulfil their duties of care in fostering research integrity better. Given the sensitivity of some of the questions, the survey will pay particular attention to ensuring the protection of the identity of the participants and their research institutions. The Randomised Response technique that will be used is expected to elicit a strong sense of trust in respondents because their answers can never be linked to them (Lentsvelt-Mulders et al. 2005). And to keep the time to complete the survey short we make use of missingness by design.

But let me be clear: surveys, focus group interviews and Delphi studies can only guide us towards potentially effective measures research institutions can take to improve responsible research practices. How good for instance SOPs and guidelines (SOPs4RI 2020) or the Hong Kong Principles (Moher et al. 2019) really are in comparison to alternative approaches needs to be sorted out in future studies designed to demonstrate effectiveness in terms of outcomes that matter.

## Conclusion

Finally it’s important to note that there are many stakeholders with a responsibility to foster research integrity. First and foremost the researchers themselves are responsible to behave well and to refrain from QRPs. Researchers should also be a good role model and help others to keep on track. Second, research institutions must empower researchers to act according to the standards of good research. But also funding agencies and scientific journals have important roles to play. There is no magic pill or a quick fix. The dilemmas and distractions researchers face are real and universal. We owe it to society to collaborate and to do our utmost best to prevent QRPs and to foster research integrity.
